# Preparing HSCT and CAR‑T therapy programmes for JACIE inspection: a structured methodological approach to implementing a quality management system

**DOI:** 10.1093/intqhc/mzag162

**Published:** 2026-10-06

**Authors:** Dimitrios Bourantas, Carlos Panizo

**Affiliations:** Department of Haematology, Hospital Universitario Donostia, San Sebastian, Spain; Department of Haematology, Hospital Universitario Donostia, San Sebastian, Spain

**Keywords:** HSCT, CAR-T, JACIE, certification, quality, inspection

## Abstract

**Background:**

Certification by the Joint Assurance Committee of the International Society for Cell & Gene Therapy and the European Society for Blood and Marrow Transplantation (JACIE) is a key mechanism for strengthening quality, safety, and organizational performance in haematopoietic stem cell transplantation (HSCT) and chimeric antigen receptor T (CAR‑T) therapy programmes. However, practical guidance on implementing a JACIE‑aligned quality management system (QMS) and preparing programmes for inspection remains limited. We present a structured methodology, developed and implemented within our programme, to support HSCT and CAR‑T therapy programmes in achieving inspection readiness while establishing a sustainable QMS that promotes continuous quality improvement.

**Methods:**

We developed and implemented a stepwise approach based on the Plan–Do–Check–Act cycle. It encompasses programme mapping and regulatory alignment, implementation of core QMS components across the collection, processing, and clinical areas, and QMS maintenance and inspection readiness, culminating in JACIE inspection with subsequent corrective actions. Key elements include process mapping, standard operating procedures, personnel competency, quality indicators, incident and deviation management, audits, validations and qualifications, product traceability, data submission, change control and risk assessment, feedback mechanisms, document management, and structured QMS review.

**Results:**

The framework was successfully implemented within our HSCT and CAR‑T therapy programme, establishing a unified JACIE-aligned QMS across the collection, processing, and clinical areas. It strengthened governance, multidisciplinary coordination, and standardization of quality management activities, providing an approach applicable to both initial JACIE accreditation and subsequent reaccreditation.

**Conclusion:**

This structured methodology provides a practical and scalable framework for implementing and maintaining a JACIE-aligned QMS in HSCT and CAR-T therapy programmes, supporting inspection readiness, multidisciplinary coordination, and the safety of patients, donors, and haematopoietic cellular therapy products. JACIE certification and its associated QMS should be viewed not as the endpoint of quality management but as part of an ongoing process of organizational learning and continuous quality improvement.

## Introduction

Haematopoietic cellular therapy (HCT) encompasses the administration of autologous or allogeneic haematopoietic cells (HCs) to restore haematopoiesis or induce specific immune‑mediated effects [1]. HCs may be infused unmanipulated or minimally manipulated, preserving their biological characteristics, or after substantial manipulation that modifies these properties [2]. The former category includes haematopoietic stem cell transplantation (HSCT), donor lymphocyte infusions, and CD34⁺‑selected grafts, whereas the latter comprises Advanced Therapy Medicinal Products (ATMPs) such as chimeric antigen receptor T (CAR‑T) cells [3]. HSCT and CAR‑T therapy represent the principal HCT modalities, indicated across a broad spectrum of diseases [4, 5]. Their complexity, spanning donor care, HCs procurement, processing and administration, and recipient management, requires robust regulatory oversight.

In Europe, HSCT is regulated by EU Directives defining safety, quality, and traceability requirements from HCs donation through distribution, including notification of serious occurrences [6–8]. CAR‑T therapies fall under the ATMP Regulation, with the Directives applying to HCs starting material donation, collection, and traceability [9]. The Regulation also governs product authorization, pharmacovigilance, efficacy monitoring, adverse reactions, and risk management, while manufacturing follows Good Manufacturing Practice (GMP) [10]. From 7 August 2027, the European Substances of Human Origin (SoHO) Regulation will replace the HSCT Directives, introducing recipient follow‑up requirements [11]. For CAR‑T therapy, the SoHO Regulation will govern donation, collection, storage, distribution, and transport of HCs starting material, while manufacturing will remain under GMP oversight [10, 11].

Within this regulatory landscape, formal certification has become increasingly important for demonstrating compliance with quality and safety expectations. In Europe, JACIE, the Joint Assurance Committee of the International Society for Cell & Gene Therapy and the European Society for Blood and Marrow Transplantation (EBMT), is the primary accreditation body for HSCT and CAR-T therapy programmes [12]. JACIE certification is based on comprehensive standards that exceed regulatory requirements and a rigorous inspection system conducted by internationally trained volunteer HCT experts [13, 14]. JACIE standards encompass all HCT phases, from donor care and HCs donation, collection, processing, preservation, distribution, and administration to recipient management. CAR‑T products are included as immune effector cells (IEC), although their manufacturing remains outside the scope of the Standards [13, 15].

JACIE promotes continuous improvement by integrating a structured quality management system (QMS) into routine practice and enabling benchmarking across centres [16, 17]. This manuscript outlines the essential steps for preparing a combined HSCT and CAR‑T therapy programme, including collection, processing, and clinical areas, for JACIE inspection through implementation of a JACIE-aligned QMS. Rather than providing an exhaustive review of the JACIE Standards, it addresses an underrepresented area in the literature by presenting a practical approach for demonstrating compliance with certification requirements. The proposed framework is intended for HSCT and CAR-T therapy programmes preparing for initial JACIE accreditation or subsequent reaccreditation.

## Materials and methods

This single-centre implementation study describes the development and establishment of a structured JACIE-aligned QMS within the HSCT and CAR-T therapy programme at Hospital Universitario Donostia. The methodological approach followed the Plan–Do–Check–Act (PDCA) cycle, comprising a 2-step preparation phase, a 13-step QMS implementation phase, and a 2-step QMS review and inspection readiness phase, culminating in JACIE inspection (Fig. 1). Inspection findings and corrective actions complete the PDCA cycle and initiate subsequent cycles of continuous quality improvement.

**Figure 1:**
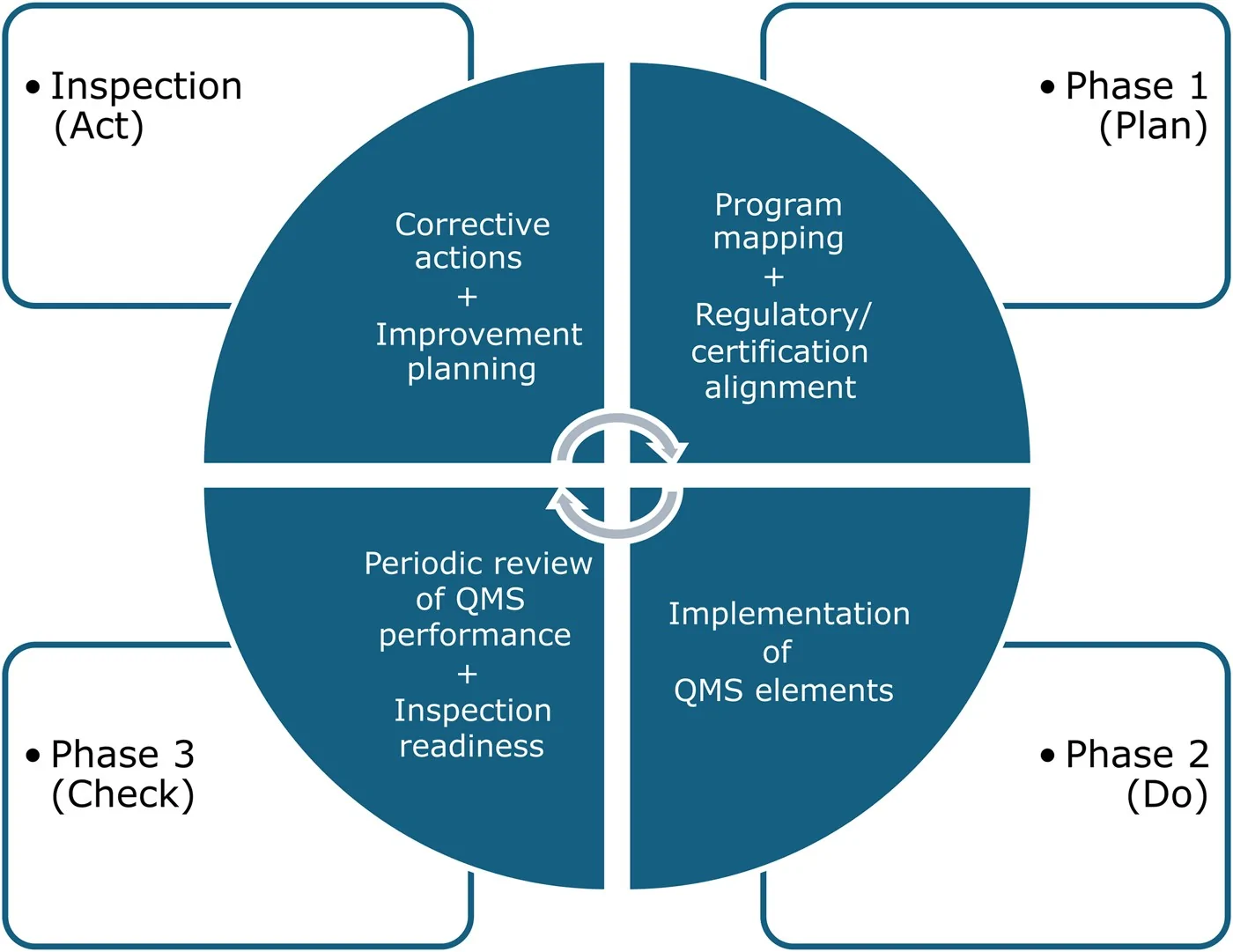
Preparing an HSCT and CAR-T therapy programme for JACIE inspection according to the PDCA cycle (deming wheel). Completion of Phases 1, 2, and 3 culminates in JACIE inspection (Act). Inspection findings inform corrective actions and improvement planning required for JACIE accreditation, completing one PDCA cycle and initiating the next to support ongoing quality improvement throughout subsequent reaccreditation cycles.

### Phase 1: initial preparation

This phase corresponds to the Plan phase of the PDCA cycle and establishes the organizational and regulatory foundations for QMS implementation.

#### Step 1: programme mapping

The first step towards JACIE inspection is systematic mapping of the programme’s organizational structure, personnel responsibilities, and interdepartmental interactions. This establishes the governance foundation for a coordinated QMS. Programmes should identify the multidisciplinary team across collection, processing, and clinical areas, including medical, nursing, technical, quality, and data management personnel, as well as key supportive specialties.

A designated quality manager (QM) should lead the preparation process with support from hospital leadership to ensure resources and institutional alignment. Organizational charts define leadership roles and reporting lines, while service level agreements with external partners, such as national donor registries and CAR-T manufacturers, are documented to ensure traceability and accountability. Structured multidisciplinary meetings with documented scope and minutes demonstrate coordinated decision-making. Facility maps of inpatient and outpatient units, Collection facilities, and Processing laboratories support understanding of programme operations and workflows.

#### Step 2: regulatory and certification alignment

A thorough understanding of the regulatory environment is essential to align internal practices with JACIE Standards. Programmes should review existing HCT‑related authorizations and applicable European and national regulations governing HSCT and CAR‑T therapy activities. The JACIE Standards and Accreditation Manual, with the latter providing detailed explanations, evidence requirements, and practical examples, should guide adaptation of workflows, policies, and documentation to ensure compliance with both regulatory and certification expectations.

Defining the certification scope is a key component of this alignment. Scope determination must reflect programme activities, including adult and/or paediatric apheresis and/or bone marrow HCs collection; cell processing with minimal or substantial manipulation; autologous and/or allogeneic transplantation; and IEC administration. Annual activity volumes must meet JACIE minimum requirements [1].

### Phase 2: QMS implementation

The second phase represents the Do phase of the PDCA cycle, during which the QMS is implemented through 13 steps. The corresponding key actions, alignment with the JACIE Standards, and expected outputs are summarized in Table 1.

**Table 1:** QMS implementation steps and corresponding key actions, alignment with JACIE standards, and expected outputs.

Implementation step	Key actions	JACIE standards alignment	Expected outputs
1. JACIE-aligned QMS	Appoint QM; ensure multidisciplinary participation	QMS governance and coordination	Harmonized QMS implementation
2. Processes and workflows	Map critical processes and responsibilities	Process integration across programme areas	Operational consistency
3. SOPs	Develop, implement and review SOPs	Documented and standardized procedures	Written instructions for programme activities
4. Personnel and training	Define roles, document qualifications, and provide training	Personnel competency and professional development	Competent and qualified personnel
5. Quality indicators	Establish, monitor and review indicators	Performance monitoring and outcome evaluation	Benchmarking and continuous improvement
6. Incidents and deviations	Report, investigate, and manage incidents and deviations	Incident/deviations reporting and corrective/preventive actions	Risk reduction and safer practice
7. Audits	Implement an audit plan and undergo external audits	Internal audits across programme areas and external audits	Continuous compliance and process improvement
8. Validations and qualifications	Validate processes and qualify equipment	Process reliability and equipment performance	Consistent and reliable procedures
9. Product labelling and traceability	Implement ISBT 128-compliant product labelling^a^	Standardized product identification	Full product traceability
10. Data submission to EBMT Registry	Ensure accurate data collection and submission	Standardized data reporting	Benchmarking and epidemiological surveillance
11. Change control and risk assessment	Manage changes and perform risk assessments	Systematic change control and risk management	Proactive risk mitigation
12. Patient and donor feedback	Collect donor and recipient perspectives	Stakeholder involvement in quality management	Quality improvement
13. Document management	Implement a document control system	Controlled QMS documentation	Secure and accessible documentation

a ISBT 128, information standard for blood and transplantation.

#### Step 1: JACIE-aligned QMS

The development and maintenance of a robust QMS is central to demonstrating compliance with JACIE standards. The QM holds primary responsibility for implementing QMS components, ensuring that quality principles are embedded across the programme [18]. Active participation from personnel in all areas is essential to support effective communication, harmonized workflows, and continuous improvement throughout HCT activities.

#### Step 2: processes and workflows

All critical HCT processes are systematically mapped according to the programme’s organizational structure. These include donor screening, testing, selection, evaluation, mobilization, and follow‑up; HCs collection, storage, transport, processing, preservation, distribution, and administration; and recipient evaluation, conditioning, post‑treatment care, and follow‑up. Each process should clearly define area‑specific responsibilities and interdependencies. Flowcharts and process diagrams help visualize these interactions and illustrate how workflows traverse all programme areas, supporting transparency and operational consistency.

#### Step 3: standard operating procedures

Standard operating procedures (SOPs) form the operational backbone of the QMS by defining how activities are performed. Their development requires involvement of relevant staff, including physicians, nurses, technical personnel, pharmacists, and intensive care unit or neurology specialists, particularly for CAR‑T‑specific workflows. Collection SOPs address donor evaluation, follow-up, mobilization, apheresis, and bone marrow harvest; Processing SOPs cover reception, manipulation, preservation, and distribution of HCT products; and Clinical SOPs describe product administration and pre‑ and post‑HCT recipient management.

Supporting documentation, including working forms, equipment and materials information, environmental parameters, sample handling, and hygiene and safety requirements, is integrated into relevant SOPs. All SOPs and associated forms must be reviewed at least every 2 years to ensure accuracy and compliance with JACIE requirements.

#### Step 4: personnel and training

Personnel competency and continuous professional development are cornerstones of a JACIE‑compliant QMS. Each staff member has a defined job description supported by documentation of professional licences, qualifications, curricula vitae, and internal and external HCT‑specific training. Competency evaluations are conducted by area Directors and supported by initial and annual training plans. The training SOP describes assessment methods, documentation requirements, and ongoing skill development processes to ensure sustained competency across programme areas.

#### Step 5: quality indicators

Quality indicators are essential for monitoring programme performance and supporting continuous improvement. They include process indicators, which assess compliance with key procedures, and outcome indicators, which reflect patient outcomes and overall programme results. Where appropriate, balancing measures may be included to identify unintended consequences of changes.

Collection indicators include mobilization efficiency, apheresis and bone marrow harvest performance, HCT product viability, and contamination rates. Processing indicators assess product viability, sterility, and integrity, while Clinical indicators include engraftment outcomes, morbidity, mortality, acute and chronic graft‑versus‑host disease, central venous catheter and nosocomial infections, and CAR‑T‑specific toxicities. Regular analysis, graphical presentation through run charts or dashboards, and dissemination of indicator results enable early detection of trends and deviations, comparison with previous performance, and informed decision-making. Review of the results supports implementation of corrective actions and continuous quality improvement.

#### Step 6: incidents and deviations

A robust system for reporting, documenting, and analyzing incidents and deviations is essential to ensure the safety of donors, recipients, and HCT products. Incidents may arise from resource limitations, organizational or infrastructure issues, equipment or material deficiencies, administrative or communication errors, training deficiencies, accidents, or human error. Reporting by all personnel is essential. Both unplanned deviations from expected practice and planned temporary deviations are recorded. The QM coordinates multidisciplinary root‑cause analyses, communicates findings to programme areas and, when applicable, external partners. Corrective and preventive actions are implemented and periodically reviewed to confirm effectiveness.

#### Step 7: audits

An annual audit plan is fundamental for verifying compliance with workflows, SOPs, and personnel qualifications. Auditors should not review activities for which they are responsible. Following audit training, personnel from one area audit activities in another area; for example, an inpatient HCT Unit nurse may audit contaminated‑product handling in the Processing area.

Internal audits are primarily process-based and encompass all programme areas. Clinical audits assess donor and patient data accuracy, EBMT Registry data submission, chemotherapy administration, and HSCT and CAR‑T toxicity management. Collection audits assess donor eligibility and suitability, while environmental monitoring audits apply to both Collection and Processing areas. Audits of external facilities, contaminated-product management, and incident follow‑up are conducted across all three areas. External audits include inspections and assessments by regulatory authorities, accreditation bodies, and commercial CAR-T manufacturers. Audit findings are disseminated across programme areas to identify strengths, weaknesses, trends, and opportunities for continuous improvement.

#### Step 8: validations and qualifications

Validations are essential to demonstrate that HCT processes consistently deliver expected outcomes under defined conditions and specified equipment, materials, and personnel. They apply to technical procedures across HCs collection, transport, reception, processing, preservation, distribution, and administration. Processing validations include HCT product testing, storage, plasma or erythrocyte reduction, freezing and cryopreservation, labelling, thawing, and preparation for administration. Collection validations cover HCs procurement, testing, labelling, storage, and distribution, while clinical validations include bone marrow harvest procedures and associated product handling. Validations are required whenever new equipment, materials, conditions, or personnel are introduced. A long-term validation plan defines periodic revalidation of procedures performed under unchanged conditions.

Equipment, materials, and reagents undergo qualification to ensure consistent performance throughout their lifecycle. A comprehensive inventory documents manufacturer and internal identifiers, storage conditions, maintenance and calibration records, expiry dates, and operational status. A robust procurement system, clear chain of custody, and organized storage areas support traceability and quality control. Contingency plans for equipment failure, including qualified backup units, ensure operational continuity.

#### Step 9: product labelling and traceability

Accurate and comprehensive labelling of HCT products is essential to ensure full traceability from donation to administration. Labelling must comply with the Information Standard for Blood and Transplantation, integrating the unique Donation Identification Number and the Single European Code. Labels must include product type, centre of origin, donation type, collection date and time, processing type, volume, additives, donor and recipient identifiers, blood group, storage and handling instructions, expiry date, and infectious-risk statements where applicable. Implementation is supported by dedicated electronic labelling software developed in collaboration with the information technology (IT) department.

#### Step 10: data submission to EBMT registry

Accurate and complete submission of HCT data to the EBMT Registry is essential for benchmarking, research, and epidemiological surveillance. Responsibility lies primarily with Data Managers in collaboration with the Clinical area. High‑quality data submission requires a reliable IT infrastructure capable of managing clinical records, capturing structured data, and archiving donor and recipient consent in compliance with data protection regulations.

#### Step 11: change control and risk assessment

Identification and evaluation of significant changes in key processes are essential to maintain operational control. Change control procedures assess the risks associated with modifications, such as changes in personnel, facilities, equipment, materials, collection or processing procedures, or clinical protocols, and define corresponding action plans and follow‑up. Programmes should also conduct periodic risk assessments of established processes to identify potential hazards, implement preventive measures, and develop contingency plans. Structured approaches, such as failure mode and effects analysis (FMEA) supported by risk registers, strengthen systematic risk identification, evaluation, and mitigation before incidents occur. Regular staff training, multidisciplinary meetings, and process reviews further support proactive risk management.

#### Step 12: patient and donor feedback

Active participation of donors and recipients in the QMS supports continuous improvement. Satisfaction surveys and formal mechanisms for reporting complaints, comments, and observations provide valuable feedback on quality of care, communication, and operational processes. Programmes may also incorporate validated patient-reported outcome measures and patient-reported experience measures to better capture patient perspectives. Survey results are analyzed and used to develop action plans that address identified issues and strengthen the QMS.

#### Step 13: document management

Implementation of all QMS components requires a secure, well‑structured document management system. This includes procedures for drafting, reviewing, approving, archiving, and disposing of documents, supported by a consistent document codification system. Close collaboration with the IT Department is essential to ensure controlled access, document protection, and data integrity. While access must be personalized and secure, critical documents should remain readily available across all programme areas to support uninterrupted clinical and operational activities.

### Phase 3: QMS review and inspection readiness

This phase corresponds to the Check phase of the PDCA cycle and focuses on reviewing and maintaining the implemented QMS and ensuring JACIE inspection readiness. Inspection findings and corrective actions constitute the Act phase, completing the PDCA cycle and initiating the next cycle of continuous quality improvement.

#### Step 1: QMS maintenance: quality meetings, annual review, and the quality manual

Regular quality meetings, coordinated by the QM and involving representatives from all programme areas, are essential for monitoring QMS performance, providing a structured forum for integrated review of all QMS elements. Their findings are consolidated into the annual QMS review report, disseminated across all areas, and used to guide improvement planning for the next PDCA cycle.

The Quality Manual serves as the programme’s central reference document. It summarizes the organizational structure, key processes, responsibilities, and inter‑area interactions, consolidating the core QMS elements and ensuring their consistent implementation and continuity across all programme areas.

#### Step 2: application for JACIE inspection

Following completion of the previous steps, the programme performs a comprehensive self-evaluation using the JACIE checklist and application form. The checklist encompasses all collection, processing, and clinical standards and requires documentation and justification of compliance with each standard. The application form summarizes the programme’s structure, activities, personnel, and systemic interdependencies, providing an overview of organizational readiness.

Both documents are submitted to the JACIE Office for initial review. Once approved, the required documentation is uploaded to the designated electronic platform. Scheduling, inspector assignment, and inspection logistics are then coordinated with the JACIE Office, ensuring readiness for the on-site inspection. Inspection findings subsequently lead to corrective actions, completing the Act phase of the PDCA cycle and informing the next cycle of continuous improvement.

## Results

The framework was implemented in our HSCT and CAR-T therapy programme ∼4 years before the JACIE inspection, with the final 2 years dedicated to routine QMS operation and monitoring. Inspection nonconformities were addressed through corrective actions, resulting in JACIE certification 1 year later. The main organizational challenge was coordinating the collection, processing, and clinical areas, whose distinct operational activities required harmonized workflows, administrative collaboration, and integrated quality management. Other challenges were primarily documentation-related, including missing or incomplete controlled documents, and were systematically resolved before inspection.

Implementation established a comprehensive organizational quality infrastructure, including more than 100 SOPs, 23 routinely monitored quality indicators, 12 internal audits, validation and qualification programmes, multidisciplinary FMEA, and an electronic incident reporting system, demonstrating successful integration of the methodology into routine programme activities (Table 2).

**Table 2:** Implementation outcomes of the proposed methodology within our HSCT and CAR-T therapy programme.^a^

PDCA phase	Methodological element	**Evidence of implementation** ^b^
Plan	Programme mapping	Organizational chart; facility maps; SLAs with REDMO and CAR-T manufacturers; HSCT, CAR-T and quality meetings
	Regulatory and certification alignment	Regulatory requirements reviewed; JACIE scope defined for apheresis/bone marrow collection, minimally manipulated processing, adult autologous/allogeneic HSCT and IEC administration
Do	JACIE-aligned QMS	QM appointed; QMS responsibilities assigned across collection, processing, and clinical areas
	Processes and workflows	Autologous/allogeneic HSCT and CAR-T workflows and flowcharts
	SOPs	Clinical (51 HSCT, 11 CAR-T); collection (8 HSCT, 2 CAR-T); processing (20 HSCT, 2 CAR-T); nursing (9 HSCT, 1 CAR-T); quality (5)
	Personnel and training	Job descriptions; competency assessments; initial/annual training records; weekly training sessions
	Quality indicators	Collection (3); processing (4); clinical (8 HSCT, 8 CAR-T) quality indicators routinely monitored
	Incidents and deviations	Electronic incident reporting system; routine incident/deviation reporting
	Audits	Twelve internal audits; 1 external CAR-T inspection
	Validations and qualifications	Process validations: collection (4); processing (6); clinical (1); equipment qualifications (20)
	Labelling and traceability	ISBT 128 and SEC implemented
	Data submission to EBMT Registry	EBMT Registry reporting; participation in EBMT benchmarking
	Change control and risk assessment	Three change control reports; multidisciplinary FMEA across all programme areas
	Patient and donor feedback	Patients/donor satisfaction surveys; hospital complaint management system
	Document management	Electronic document management system; controlled access; internal document codification
Check	Quality meetings, annual review and the quality manual	Weekly quality meetings; annual QMS review report; quality manual established
	Application for JACIE inspection	JACIE self-assessment checklist completed; inspection application accepted by JACIE office
Act	–	JACIE inspection; corrective actions implemented; certification obtained

a The proposed methodology culminates in JACIE inspection; therefore, no separate methodological element is defined for the Act phase. —, not applicable.

b ISBT 128, information standard for blood and transplantation; REDMO, Spanish Bone Marrow Donor Registry; SEC, single European code; SLA, service level agreement.

## Discussion

### Statement of principal findings

The methodological model presented provides a structured, phased approach for implementing the JACIE Standards within HSCT and CAR-T therapy programmes through the establishment of a unified QMS across the collection, processing, and clinical areas. It follows the PDCA cycle, with the Plan, Do, and Check phases culminating in JACIE inspection and subsequent corrective actions completing the cycle and driving continuous quality improvement. Implementation within our programme demonstrated the framework’s feasibility and resulted in measurable organizational outcomes.

### Strengths and limitations

A major strength of the model is its practicality, providing a scalable approach for implementing a unified JACIE-aligned QMS across the collection, processing, and clinical areas instead of treating them as independent branches. Its design makes it applicable to both HSCT and CAR-T therapy programmes while facilitating the integration of new techniques, procedures, and equipment within an established QMS.

However, the methodology was developed and implemented within a single programme, requiring external validation in different organizational settings to confirm its generalizability. Adaptation according to programme size, maturity, and available resources may also be required.

### Interpretation within the context of the wider literature

Evidence demonstrates that JACIE-aligned QMS implementation improves HSCT outcomes [19, 20] and donor management practices [21, 22]. However, the existing literature provides limited operational guidance on implementing the JACIE Standards and preparing programmes for JACIE inspection. To our knowledge, this is the first study to present a comprehensive, stepwise methodology for implementing a JACIE-aligned QMS across the collection, processing, and clinical areas of combined HSCT and CAR-T therapy programmes. The model complements the published literature by providing a practical, process-oriented approach to QMS implementation based on quality management principles and the PDCA cycle.

### Implications for policy, practice, and research

JACIE certification has become increasingly important for demonstrating compliance and quality in HSCT and CAR-T therapy activities [14, 15, 23]. However, certification should not be regarded as the ultimate goal of quality management. While quality assurance ensures adherence to defined standards, a mature QMS extends beyond compliance by promoting continuous quality improvement through performance monitoring, risk management, and continuous learning [24]. By embedding JACIE-aligned QMS elements within the PDCA cycle, the proposed approach integrates certification into an ongoing system that supports a culture of quality and safe HCT delivery.

Establishing a JACIE-aligned QMS is a complex organizational process, requiring collaboration across programme areas, leadership and staff engagement, multidisciplinary teamwork, communication, and organizational readiness. Our framework facilitates implementation through clear allocation of responsibilities and regular quality meetings that promote shared understanding of processes. By integrating collection, processing, and clinical areas, it enhances coordination within the programme and with external stakeholders, including donor registries and CAR-T manufacturers. Collaboration with experienced JACIE-certified centres and existing hospital quality frameworks may further facilitate adoption. Based on core QMS principles rather than specific regulatory requirements, the methodology can adapt to evolving regulations, including the forthcoming SoHO Regulation, without fundamental redesign.

The proposed approach supports the safety of patients, donors, and HCT products. Standardized procedures, validated processes, product labelling and traceability, incident and deviation management, risk assessment, and corrective actions help preserve product integrity while ensuring safe donor and patient care throughout the HCT pathway. By embedding these activities within routine practice, the framework promotes a proactive safety culture focused on continuous improvement.

As QMS continue to evolve towards more integrated and data-driven approaches [25], future research should evaluate the proposed methodology across programmes of different sizes, levels of maturity, and organizational contexts to determine its generalizability. Further studies should explore how digital QMS platforms can support implementation and long-term maintenance of the model through enhanced document control and real-time performance monitoring. Such approaches may facilitate adaptation to emerging HCT therapies and evolving regulatory requirements while maintaining high standards of quality and safety.

## Conclusions

Preparation for JACIE certification is a demanding process requiring coordination across all HCT programme areas. The structured methodology presented provides a practical and scalable framework for implementing, maintaining, and continuously improving a unified JACIE-aligned QMS within combined HSCT and CAR-T therapy programmes, supporting inspection readiness, stakeholder collaboration, and the safety of patients, donors, and HCT products. As HCT continues to evolve, JACIE certification and its associated QMS should be viewed not as the endpoint of quality management but as part of a dynamic process of organizational learning and continuous improvement.

## Data Availability

No data sets were generated or analyzed for this study. Data sharing is not applicable.
